# 4347 Bimodal Visual-Olfactory Training for Post-Surgical or Post-Traumatic Olfactory Dysfunction (VOLT Trial)

**DOI:** 10.1017/cts.2020.110

**Published:** 2020-07-29

**Authors:** Andrew Michael Peterson, Dorina Kallogjeri, Jay Piccirillo

**Affiliations:** 1 Washington University School of Medicine

## Abstract

OBJECTIVES/GOALS: 1) Assess the patient-reported, perceived change in olfactory function after bimodal visual-olfactory training (OT) 2) Assess change in olfactory function after bimodal visual-olfactory training with a smell identification test 3) Assess which scents are most important to people with olfactory dysfunction (OD) METHODS/STUDY POPULATION: The participants are adults with subjective or clinically diagnosed OD with post-surgical or traumatic etiologies within the last 5 years. At the first of two study visits, participants complete the University of Pennsylvania Smell Identification Test (UPSIT) and complete general health (SF-36) and olfactory-related quality-of-life questionnaires. From a list of 34 scents, participants chose the 4 scents most important to them and smelled the scents twice daily for 3 months. Olfactory testing and the quality-of-life questionnaires were repeated at the final visit. RESULTS/ANTICIPATED RESULTS: 10 participants have enrolled in the study. There was one screen fail and one withdrawal. Six participants are currently undergoing OT and two have completed the study. Seven participants have post-surgical etiology and three have post-traumatic etiology of their OD. Of the two participants who have completed the study, one had an UPSIT score improvement from 25 to 33 out of the 40 questions correct. The minimally clinically important difference on the UPSIT is 4. She reports improvement subjectively. The second participant had a UPSIT score change from 25 to 24 and reports ability to smell is neither better nor worse. DISCUSSION/SIGNIFICANCE OF IMPACT: Traumatic and post-surgical, particularly post-transphenoidal hypophysectomy, are common etiologies of OD and no effective treatments exist. The results from our pilot study will help better inform the best way to undergo OT, how effective it is, and the planning of future studies.

